# Zika virus triggers autophagy to exploit host lipid metabolism and drive viral replication

**DOI:** 10.1186/s12964-022-01026-8

**Published:** 2023-05-19

**Authors:** Gloria Stoyanova, Sidra Jabeen, Joselyn Landazuri Vinueza, Sounak Ghosh Roy, Richard A. Lockshin, Zahra Zakeri

**Affiliations:** 1 Department of Biology, CUNY Queens College, Flushing, NY 11367 USA; 2 Department of Biological Sciences, St. John’s University, Jamaica, NY 11439 USA; 3 Present Address: Cellular and Molecular Biology, University of Wisconsin-Madison, Madison, WI USA; 4 Present Address: Department of Genetics, Albert Einstein College of Medicine, Bronx, NY 10461 USA; 5 Present Address: Department of Microbiology, University of Washington, Seattle, WA 98195 USA; 6 Division of Human Biology, Fred Hutchinson Cancer Research Center, Seattle, WA 98109 USA; 7 Present Address: Henry Jackson Foundation for the Advancement of Military Medicine, Silver Spring, MD 20910 USA

**Keywords:** Zika virus, Autophagy, Lipid droplets, Atorvastatin, Statins, Viral replication

## Abstract

**Background:**

Zika virus (ZIKV), an arbovirus of global concern, has been associated with neurological complications including microcephaly in newborns and Guillain–Barré syndrome in adults. Like other flaviviruses, ZIKV depends on cholesterol to facilitate its replication; thus, cholesterol has been proposed as a therapeutic target to treat the infection using FDA-approved statins. Cholesterol is stored in intracellular lipid droplets (LD) in the form of cholesterol esters and can be regulated by autophagy. We hypothesize that the virus hijacks autophagy machinery as an early step to increase the formation of LD and viral replication, and that interference with this pathway will limit reproduction of virus.

**Methods:**

We pretreated MDCK cells with atorvastatin or other inhibitors of autophagy prior to infection with ZIKV. We measured viral expression by qPCR for NS1 RNA and immunofluorescence for Zika E protein.

**Results:**

Autophagy increases in virus-infected cells as early as 6 h post infection (hpi). In the presence of atorvastatin, LD are decreased, and cholesterol is reduced, targeting key steps in viral replication, resulting in suppression of replication of ZIKV is suppressed. Other both early- and late-acting autophagy inhibitors decrease both the number of LD and viral replication. Bafilomycin renders cholesterol is inaccessible to ZIKV. We also confirm previous reports of a bystander effect, in which neighboring uninfected cells have higher LD counts compared to infected cells.

**Conclusions:**

We conclude that atorvastatin and inhibitors of autophagy lead to lower availability of LD, decreasing viral replication. We conclude that bafilomycin A1 inhibits viral expression by blocking cholesterol esterification to form LD.

Video Abstract

**Graphical Abstract:**

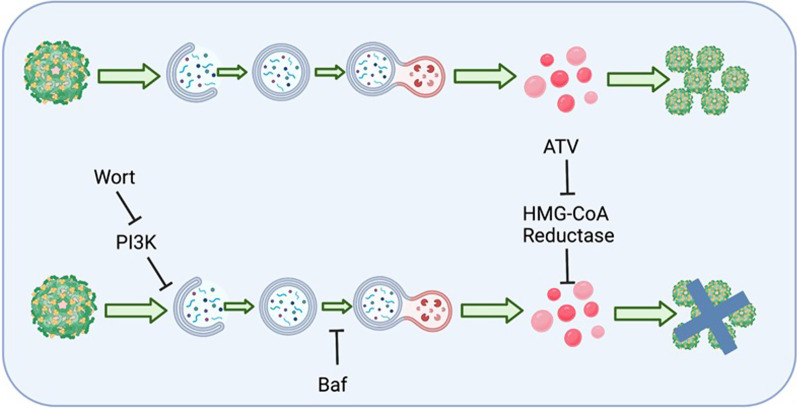

**Supplementary Information:**

The online version contains supplementary material available at 10.1186/s12964-022-01026-8.

## Background

ZIKV is an emerging mosquito-borne, positive-sense single-stranded RNA virus belonging to the family *Flaviviridae* [1]. Since the discovery of ZIKV in 1947 [2], ZIKV infections in humans have been generally asymptomatic, sporadic, and limited to Africa and Asia. ZIKV began to erupt outside Africa and Asia in 2004, with more than 80 countries currently reporting ZIKV transmission [3]. These outbreaks led to an increase in devastating birth defects [4, 5] and Guillain–Barré syndrome in adults [6, 7] highlighting the importance of understanding host cellular pathways that the virus exploits during the course of infection.

ZIKV carries out each stage of its replication cycle in close association with cellular membranes within specialized replication complexes formed from extensively remodeled ER membranes [8]. These serve as sites of lipid biosynthesis, including cholesterol, sphingolipids, and ceramides [9]. Viral replication depends on the metabolism of lipids and cholesterol [10], the latter of which accounts for 11–12% of their total mass [11].

ZIKV generates large numbers of lipid droplets (LD), dynamic ER-derived organelles that are exploited by positive sense RNA viruses to acquire lipids for membrane or energy production to support their replication [12]. We investigated how ZIKV perturbs LD formation to promote infection.

LD are consumed by autophagy under stress conditions. The increase in autophagy in infected cells, engulfment of LD in autophagosomes, and enrichment of LD within autophagolysosomes suggests that autophagy in infected cells contributes to the increase of LD through lipophagy [13], possibly increasing ATP via β-oxidation through a still uncharacterized regulatory mechanism [12]. Autophagy is important for the host defense against viral infection [14, 15]. However, viruses have evolved some mechanisms to modulate autophagy to increase viral survival and replication. Some RNA viruses, such as influenza A virus [16], dengue virus [17], hepatitis C virus [18] and ZIKV [19], induce autophagosome formation to promote viral genome replication.

We hypothesized that ZIKV infection dysregulates formation of LD by targeting autophagy and lipid formation to promote viral replication. Here, we demonstrate that this hypothesis is correct. Inhibition of LD by atorvastatin (ATV), a reversible inhibitor of 3-hydroxy-3-methylglutaryl-CoA (HMG-CoA) reductase, reduces formation of LD and replication of ZIKV, as does inhibition of autophagy by bafilomycin A1 (an inhibitor of autophagosome-lysosome fusion). These results show that LD availability plays an important role in ZIKV replication.

## Materials and methods

### Isolation, culture, and titration of ZIKV

Vero and Vero E6 cells were seeded at 3 × 10^6^ cells in 75 T flasks and allowed to attach overnight. Cells were infected with ZIKV-MR766 (ATCC® VR-84™) at a MOI (Multiplicity of Infection) of 0.1 for 2 h (h); then cells were covered with DMEM with 2% FBS. After three days at 37 °C and a humidified atmosphere, the supernatant was collected, and cell debris was separated by centrifugation at 2000 rpm at 4 °C for 10 min. The supernatant, containing mature virions, was collected, aliquoted, and stored at – 80 °C.

The viral titer was then determined by the traditional plaque assay as follows: Vero E6 cells were suspended and approximately 2.5 × 10^5^ cells were allowed to attach overnight in 12 well plates, in DMEM supplemented with 10% FBS and 1% penicillin. The following day, confluent monolayers were infected with tenfold serial dilutions of virus suspension and the virus was permitted to attach for 2 h at 37 °C. Infected cells were rinsed once with 1X PBS and then covered with the agar overlay, containing 50% low melting point agar (# V2111, Promega), 40% 2X DMEM and 10% FBS. The agar overlay was allowed to solidify at room temperature (RT), and the cells were incubated for five days at 37 °C to facilitate plaque development. Before plaque count, cells were fixed with 4% formaldehyde (# F8775, Sigma) for 20 min. The solidified agar was removed, and cells were washed with 1X Phosphate Buffered Saline (PBS) and stained with a 1% crystal violet solution (# C0775, Sigma) for 10 min. Plaques were counted, and the virus titer was expressed as PFU/ml.

### Cell culture and treatments

MDCK (Madin-Darby Canine Kidney Epithelial Cells, ATCC®-CCL-34™), Vero (African green monkey kidney epithelial cells, ATCC® CCL-81™), and Vero E6 cells (ATCC^©^ CRL-1586™) were purchased from the American Type Culture Collection (ATCC). MDCK, Vero and Vero E6 cells were grown in Dulbecco's modified Eagle medium (DMEM, catalog # 12800017, Invitrogen) supplemented with 10% heat-inactivated fetal bovine serum (FBS, Thermo Fisher, # 16000044) and 1% penicillin–streptomycin (PS, Invitrogen, # 15140122). All cell lines were incubated at 37 °C under a humidified atmosphere consisting of 5% CO_2_ and 95% air.

As described in Ghosh Roy et al. [20], cells were seeded at 2.5 × 10^5^ cells per well in a 6-well plate and allowed to attach overnight. The next day, ZIKV MR766 (ATCC® VR-1838TM) was added to the cells at an MOI of 1, and the cells were incubated for 2 h before washing with and adding fresh media. The infected cells were further cultured for 12, 24, or 48 h depending on what we were testing. However, most of the data presented represent experiments terminating at 48 h. We also examined the role of the PERK pathway by exposing cells to class I/III PI3K inhibitor Wortmannin (wort, #681675, Calbiochem) at 50 μM, HMG-CoA reductase inhibitor Atorvastatin (ATV, #Y0001327, Sigma) at 5 μM, Salubrinal (Sal, #sc-202332, Santa Cruz) at 3 μM, or Tunicamycin (Tunica, #T7765, Sigma) at 3 μM. In all these cases, cells were incubated with inhibitors for 1 h prior to infection.

For certain experiments cells were treated with 50 nM bafilomycin A1, a vacuolar H^+^-ATPase (V-ATPase) inhibitor used to inhibit autophagy, either before or after infection. For bafilomycin pretreatment, cells were seeded as described above and treated the next day with 50 nM bafilomycin. After one hour the medium was aspirated and the cells were incubated with ZIKV at MOI 1 for 2 h. After the incubation period was over, fresh cell culture media was added to the cells and allowed to grow for 12, 24 or 48 h.

### Immunofluorescence

Immunofluorescence was performed as described in Lin et al. [21]. MDCK were grown on glass coverslips to 70% confluence. Appropriate treatments were done as described above. At the indicated endpoint of each treatment, cells were washed twice with 1X PBS, and fixed with 4% paraformaldehyde for 1 h at RT. Cells were permeabilized with 0.2% Triton X-100 (#X100, Sigma) in 1X PBS for 15 min at 37 °C. Then, cells were washed three times with 1X PBS for 5 min each. Cells were treated with 1% BSA (# A2153, Sigma) 0.1% Triton X-100 in 1X PBS for 1 h before addition of antibodies. Cells were incubated overnight at 4 °C with various antibodies depending on the experimental needs. To check for the presence of Zika infection a Anti Zika E-protein rabbit polyclonal Ab was used at a dilution of 1:200 (# Ab00230-23.0, Absolute antibody) or a viral E protein mouse monoclonal antibody (isolated from Hybridoma cells, ATCC® HB-112) with 1:10 dilution. To stain for LC3 puncta Anti-LC3B polyclonal rabbit Ab was used at a dilution of 1:100 (#L7543, Sigma). Other antibodies used and the respective concentrations can be found in Additional file 1: Table. Following overnight incubation, cells were washed three times with 1X PBS for 5 min. Cells were incubated with appropriate secondary antibodies for 1 h at room temperature at a dilution of 1:1000 (see Additional file 1: Table). They were then washed with 1X PBS for 5 min and then stained with 4, 6 -diamidino-2-phenylindole (DAPI) (1 mM) (# ab228549, ABCAM) for 8 min. Cells were washed twice with 1 × PBS, mounted, and embedded in Fluoromount® and observed at 40X and 100X by fluorescence microscopy using the Leica Leitz DMRB microscope. Details of all antibodies are described in Additional file 1: Fig. 6S.

### Filipin staining

Filipin III was dissolved in anhydrous dimethylformamide under inert gas conditions, to form a stock concentration of 1 mg/ml. Filipin was diluted 1:20 to 0.05 mg/ml in PBS and added to cells with the secondary antibodies on day 2 of immunofluorescence in complete darkness for two hours.

### Lipid droplet staining and quantification

For measurement of lipid generation, cytological analysis of LD using Oil Red O (ORO) (# O0625, Sigma), a fat-soluble dye that stains lipids, was performed as described previously [22].

Briefly, after treatment and fixation, cells on coverslips were washed with 60% isopropyl alcohol and then dried for a few hours or overnight. Staining can be performed alone or during immunofluorescence after the secondary antibody incubation. LD in samples were then stained with 60% ORO solution for 20 min and coverslips were rinsed four times with distilled water. Samples were then mounted on glass slides with Fluoromount® and visualized with the same fluorescence microscope. The total red fluorescence per cell was quantified using ImageJ software. (ImageJ, along with its updated version Fiji, is a free yet powerful image analysis/statistics package developed by personnel of the National Institutes of Health (USA), downloadable at ImageJ (nih.gov). The numbers were reported calculated by multiplying the mean fluorescence of the cell by the area of the cell. This was done for every cell in each frame. Their averages were reported as mean total ORO/cell in arbitrary units. At least 200 cells from different sections of a given slide were used in our quantification. Each experiment was done at least 3 times. Examples of how the quantification was done are found in Additional file 1: Fig. 5S.

### Quantitative RT-PCR

Cell lines were infected and treated as described above. According to the manufacturer’s protocol, total mRNA was isolated from mock-infected and ZIKV-infected cells with the GenElute™ Total RNA Purification Kit (Sigma, # RNB100). Power SYBR™ Green RNA-to-Ct™ 1-Step Kit (catalog no. 4391178, Thermo Fisher) was then used to reverse-transcribe and obtain the cDNA, followed by real-time PCR. The following primers were used to quantify the target gene (ZIKV-NS1) and the loading control (beta-tubulin):

NS1 forward primer 5′ TACACCCAGTCACAATAGGAGAGTG 3′/reverse primer 5′ CCATGCATTCATTGTCACACTTGTGG 3′, tubulin forward primer 5′ AGGATTCGCAAGCTGGCTG 3′/reverse primer 5′ TAATCCACAGAGAGCCGCTCC 3′.

### Statistical analysis

The total fluorescence for LD reported was analyzed using ImageJ as reported previously [22]. Briefly, a random field of cells was selected. Each cell within the field was circled and analyzed for the area of the cell and the intensity of the red channel (LD channel). The product of these values gave a quantity for each cell which was averaged for each condition and compared between experimental conditions. Their averages were reported as mean total ORO/cell in arbitrary units.

Statistical significance of the results was calculated by two sample unequal variance *t* test using a two-tail distribution through excel; values of *P* < 0.05 represent no statistical difference between compared samples. Pearson Correlation Coefficient was calculated using excel. The average of each time point (12 h, 24 h, 48 h) was taken for Normalized Ct values and Average ORO as a measure of lipid quantification, and these values were used to generate the Pearson Correlation Coefficient in Additional file 1: Fig. 1SC.

## Results

### Zika infection changes host LD in a time dependent manner

**Fig. 1:**
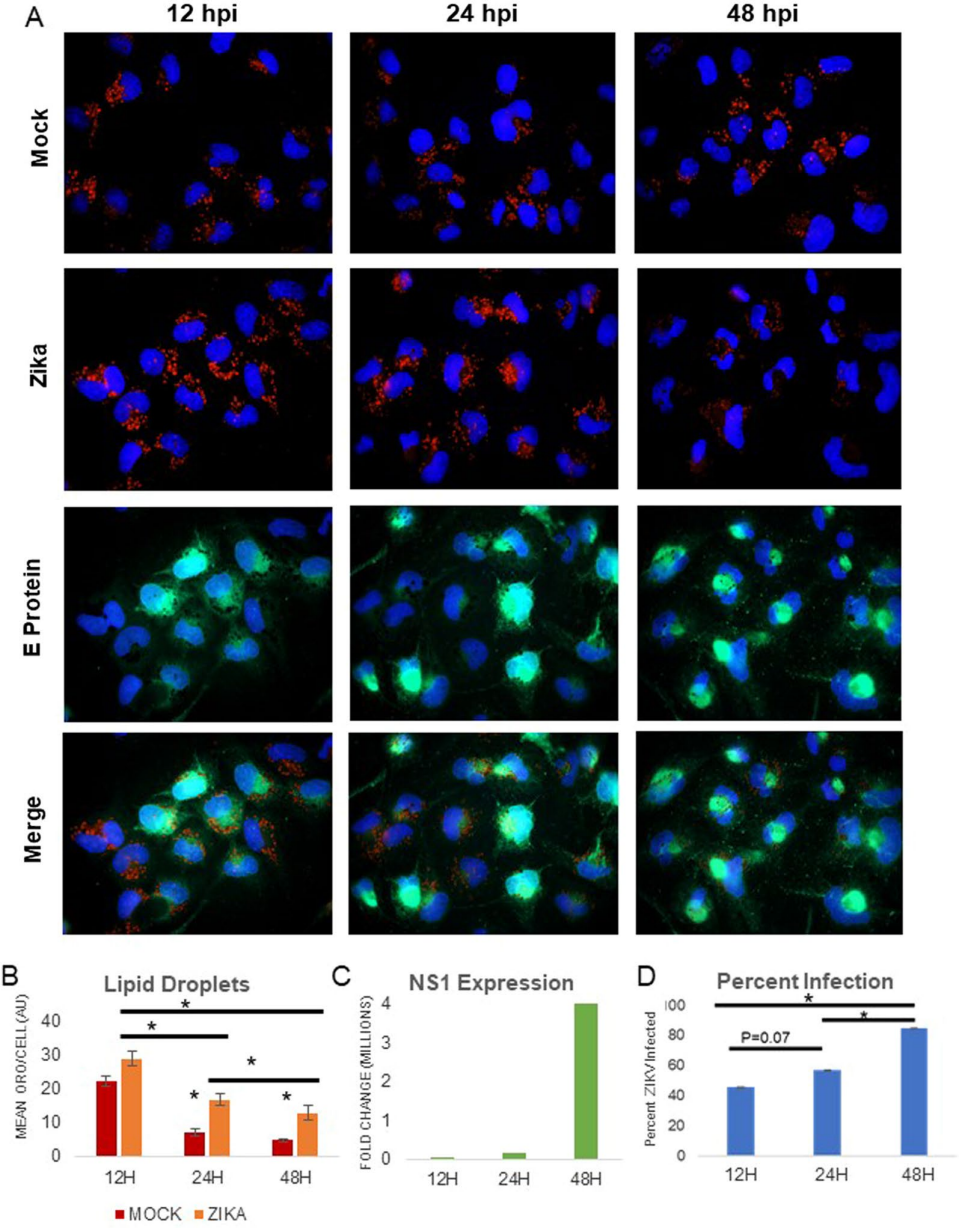
Zika infection changes host LD in a time dependent manner. LDs are stained red (Oil Red O) particles in both mock- and Zika-infected cells after 12, 24, and 48 h of infection (MOI 1). **A** MDCK cells were infected with Zika virus at MOI of 1 and analyzed at the indicated time point. While cells in culture accumulate some LDs even if they are not infected (mock), infected cells show a gradual increase in lipid droplets, followed by LD depletion as time progresses. Images shown here are representative of at least three independent experiments. **B** LD quantification using ImageJ being measured as total red fluorescence per cell. Lipid accumulation is significantly increased at 24 and 48 h, though at 12 h the p value = 0.07. LD quantifications were done by analyzing more than 200 cells for each condition. **C** Production of viral RNA as detected by qPCR and is expressed as fold change over mock NS1 expression. There is more viral RNA at 48 H compared to 12 and 24 H. Similar fold change values were obtained from three independent experiments. Correlation between lipid droplets and viral RNA is presented in Additional file 1: Figure S1. **D** Percent infection (100 × (infected cells/total cells))

We evaluated the amount of LD fluorescence in cells infected with ZIKV at MOI 1 at 12, 24 and 48 h post-infection (hpi). Infectivity was measured by detecting the expression of the ZIKV envelope protein (E protein) using an anti-E protein antibody for immunofluorescence (Fig. 1A, D). E protein expression was highest at 48 hpi. We quantified the change of LD by measuring total Oil Red O (ORO) fluorescence per cell using fluorescence microscopy and computer software. As early as 12 h, upon completion of a full replication cycle for the virus, LD are upregulated compared to mock-infected cells (mock) (*p* = 0.07) (Fig. 1A, B). Increases in LD compared to mock were seen at all three times, with the number of droplets decreasing with time (Fig. 1A, B) suggesting the overall consumption or exhaustion of LD as viral titer increases (Fig. 1C). This relationship between LD and ZIKV NS1 expression is reflected in our Pearson correlation coefficient of 0.97 (Additional file 1: Fig. 1SC). Overall, these data highlight the dynamic redistribution of host lipids by ZIKV and suggest that Zika may deplete lipid stores in later stages of infection.

### Zika virus upregulates lipids in bystander cells

While the response of individual cells was variable, we noticed that cells adjacent to infected cells contained more LD than infected cells at 48 h. Quantification of the average ORO/cell (indicated that these neighboring cells (ZIKV E-) contained more LD than zika infected cells (ZIKV E+) (Additional file 1: Fig. 1S; *p* value < 0.001). In cultures of infected cells, both ZIKV E+ and ZIKV E- cells had more LD compared to mock (Additional file 1: Fig. 1SB). This result suggests utilization and exhaustion of LD in infected cells compared to their uninfected counterparts or a bystander effect, driven either by excreted metabolic products from infected cells or these latter draining substrates from the local environment.

### Atorvastatin reduces virus induced LD availability and inhibits ZIKV production

**Fig. 2:**
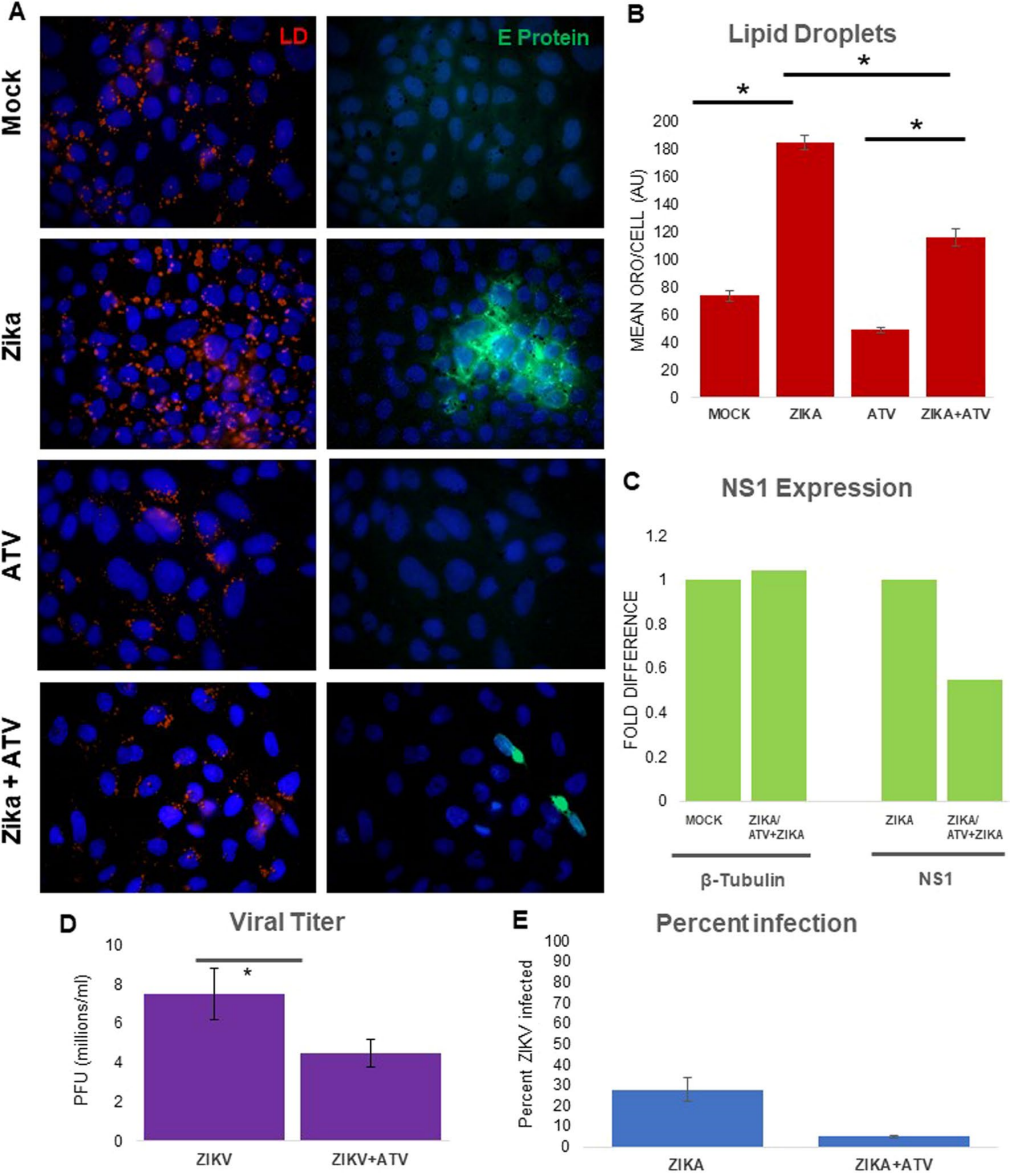
Atorvastatin reduces virus induced lipid droplet formation and inhibits zika virus production. **A** MDCK cells were pretreated with 5 um ATV for 1 h before infection. Cells were infected with ZIKV at MOI 1 for 48 h. ATV treatment reduces LD in infected cells almost to the amount seen in mock-infected cells (Compare Zika to Zika + ATV) and this difference is significant (B). ATV treatment also partially blocks replication of the virus. ZIKV E protein is immunofluorescent (green), lipids (red) and nuclear DNA (blue). **B** Image J quantification of the LD in each condition. ATV reduces LD in infected cells to levels almost seen in mock-infected cells. **C** Production of viral RNA as measured by qPCR is shown as fold change over mock NS1 expression. Viral NS1 RNA decreases in the presence of ATV. **D** A plaque assay was done on ATV pretreated infected cells and infected cells alone at MOI 5. (Lower infection was necessary for the microscopy.) ATV pretreatment reduces production of mature virus. **E** % Infection was calculated by counting infected cells/ total cells and multiplying by 100. These results confirm the finding in panel A that ATV treatment also partially inhibits production of Viral E protein

ZIKV increases the number of LD. We considered that interfering with formation or metabolism of LD could limit viral growth. We therefore exposed ZIKV-infected cells to ATV (which inhibits the biosynthesis of cholesterol) and then measured LD and NS1 RNA. We pretreated MDCK cells for 1 h with 5 μM ATV, followed by Zika infection (MOI 1) for 2 h. ATV reduced availability of LD in Zika-infected cells to that seen in mock-treated cells (Fig. 2A, B). Treatment with ATV decreased NS1 transcription by approximately 50% (Fig. 2C) as well as the expression of E protein (Fig. 2A) compared with infection alone. We also performed a plaque assay using supernatant from MDCK cells infected with ZIKV at MOI 5 and found that pretreatment with ATV significantly reduced the release of mature virions (Fig. 2D). To visualize how cholesterol, a major class of host lipids and the target of ATV, is reorganized during ZIKV infection we used filipin III, which stains unesterified cholesterol (Additional file 1: Fig. 2S). In untreated cells, cholesterol is distributed throughout the cell. In infected cells, cholesterol is localized to the sites of viral infection. Zika-infected cells pretreated with atorvastatin show reduced cholesterol localization to the plasma membrane. Thus, based on these results, the inhibition of cholesterol synthesis by ATV can seriously decrease LD and ZIKV replication.

### Zika infection induces autophagy and inhibition of autophagy decreases LD

**Fig. 3:**
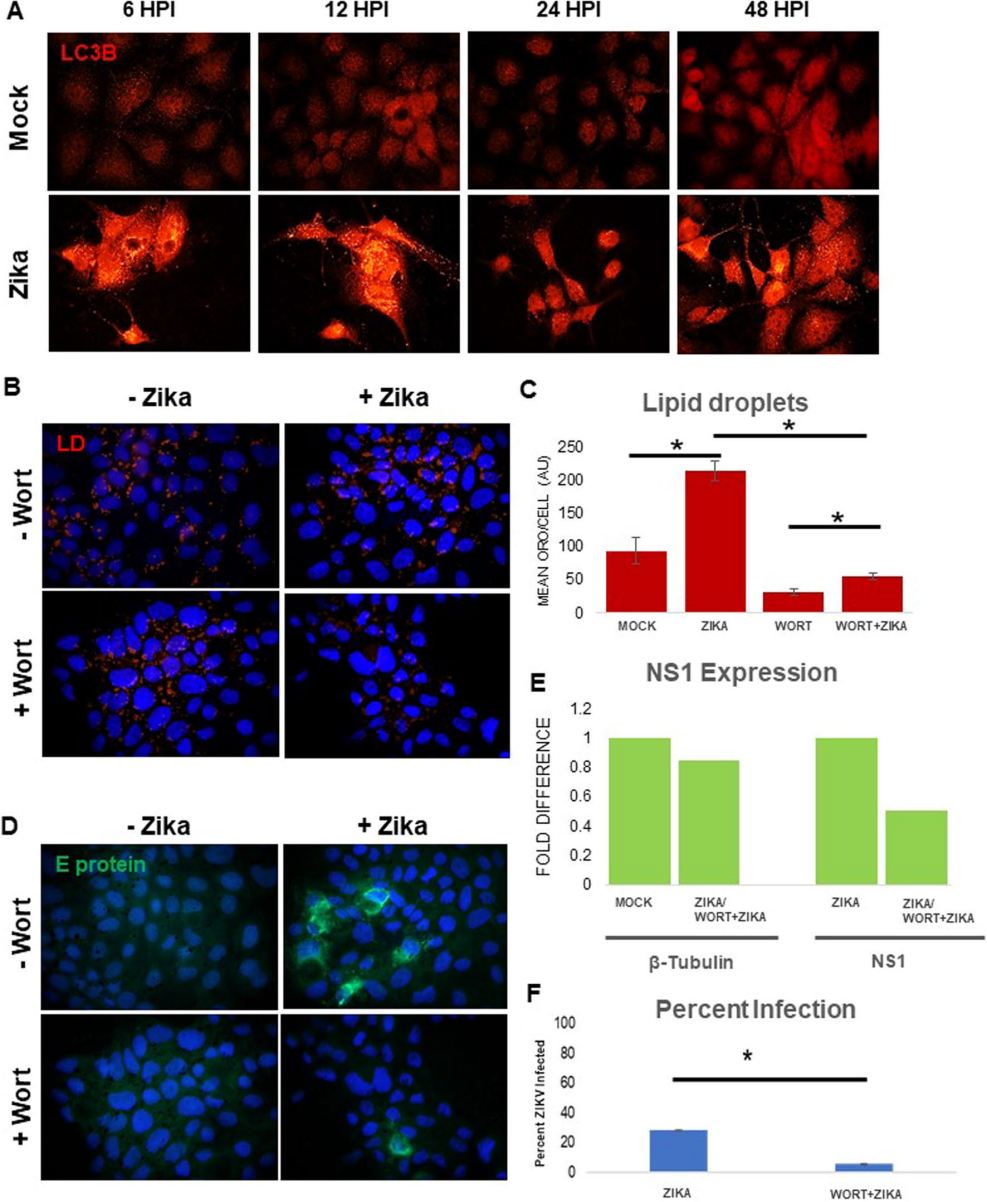
Zika infection induces autophagy and inhibition of autophagy decreases lipid droplets. **A** MDCKs were infected with ZIKV at MOI 1 for 6, 12, 24 and 48 h. Immunofluorescence for LC3 was used as a marker for induction of autophagy. More than minimal punctation was never seen in mock-infected cells. Activation of autophagy, seen early during infection at 6 and 12 h as evidenced by bright punctation, drops by 24 h (less puncta) and is restored to initial levels by 48 h. **B** MDCKs were pretreated with autophagy inhibitor wortmannin at 50 mM, 1 h before infection at MOI 1 for 48 h. Lipid droplets were stained with ORO during immunofluorescence. Wortmannin partially blocks the induction of lipid droplets by zika (Compare Zika + wort to Zika). Viral replication is also decreased in the presence of wortmannin as detected by immunofluorescence. **C** This reduction in lipid droplets is statistically significant as analyzed by ImageJ (Compare wort + Zika to wort). Quantifications are analyzed in more than 200 cells for each condition. **D** MDCKs were pretreated with autophagy inhibitor wortmannin at 50 mM, 1 h before infection at MOI 1 for 48 h. Viral replication was measured by using an Anti-E protein primary antibody (green). Wortmannin reduced the production of Zika E protein (Compare Zika to Wort + Zika). **E** qPCR measuring ZIKV NS1 mRNA was performed to verify the effects of ATV on viral replication. ATV pretreatment reduced ZIKV replication approximately 50%. **F** Quantification of ZIKV E protein expression shows statistically significant decrease %) in infection following wortmannin treatment. Percentage of infection. calculated as 100 x (cells expressing ZIKV E protein (green/total cells)

Rapid LD biogenesis has been detected after infection with influenza A, Dengue virus, and Hepatitis C viruses. Relatively little is known regarding the molecular mechanisms that regulate LD formation during ZIKV infection; some authors favor the hypothesis of autophagy activation leading to lipid droplet accumulation [23]. Thus, we monitored autophagy through the autophagy marker LC3-II in ZIKV infected cells at 6, 12, 24, and 48 hpi. During autophagy, cytosolic LC3-I is lipidated to form LC3-II, which associates with autophagosome membranes and facilitates the recruitment of cargo into the pathway; thus, an increase in the LC3-II is indicative of induction of autophagy. Autophagy was activated at 6 and 12 hpi, decreased at 24 hpi, and rose again at 48 hpi (Fig. 3A). Thus, ZIKV infection activates autophagy. We considered how LD would change if autophagy were blocked. MDCK cells were pretreated with the autophagy inhibitor (PI3K inhibitor) Wortmannin, for one hour prior to infection at MOI 1. Inhibition of autophagy reduces the formation of LD in infected cells (*p* value < 0.001) (Fig. 3B, C). Infected cells treated with autophagy inhibitors decreased viral NS1 transcription by 50% (Fig. 3E), while percent infection was reduced approximately 80% (Fig. 3D, F). Taken together, our data suggest that induction of autophagy may play a role in lipid upregulation and contributes to viral replication.

### Bafilomycin A1 disrupts cholesterol trafficking in zika infected cells

**Fig. 4:**
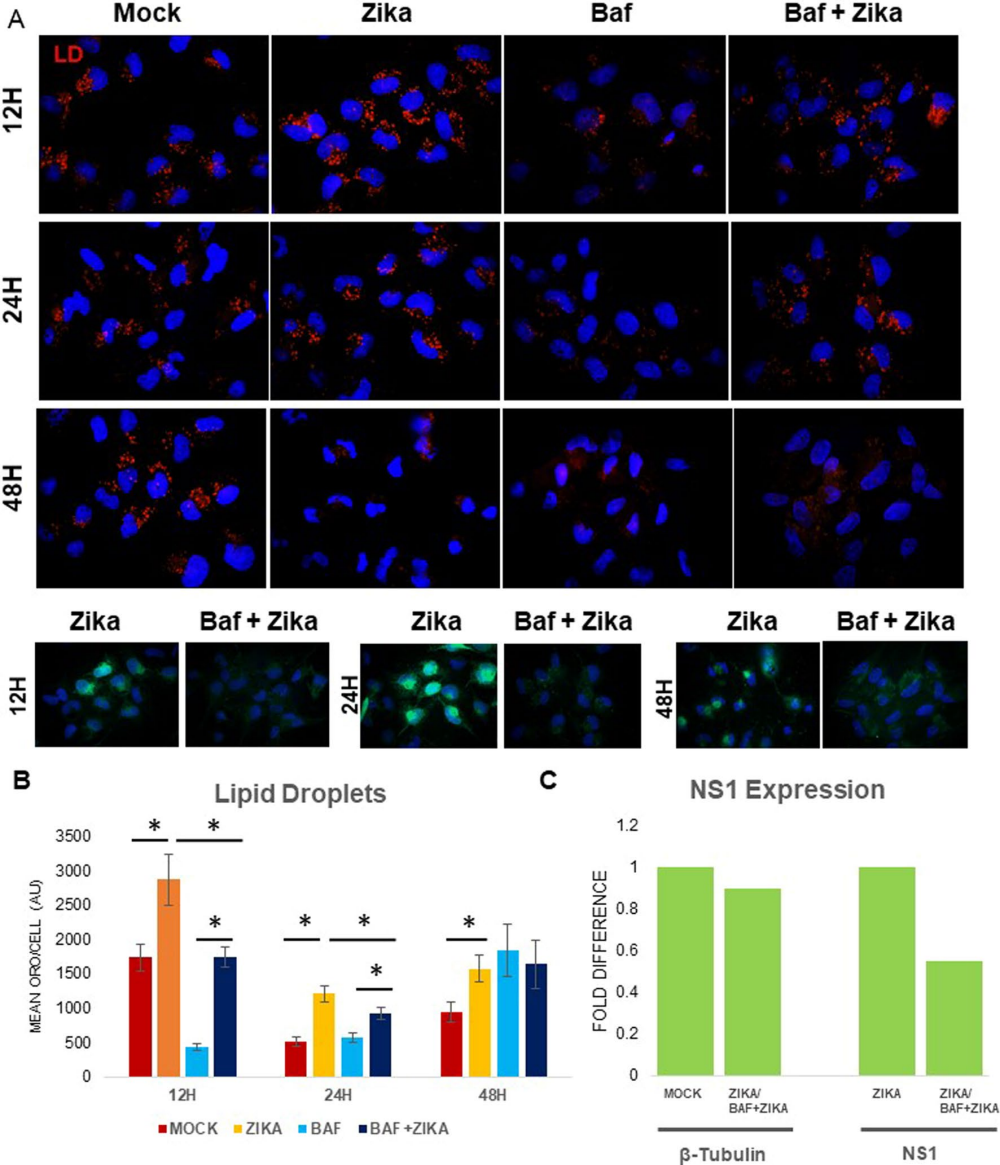
Bafilomycin A1 inhibits viral expression and lipid formation. **A** MDCK cells were infected at MOI 1 for 12, 24 and 48 h with and without pretreatment with 50 nM Bafilomycin, an inhibitor of autophagy. Immunofluorescence was performed using primary antibody against ZIKV E-protein (green) and ORO to stain LD (red). Bafilomycin pretreatment significantly reduced LD and Bafilomycin pretreated Zika infected cells have little to no detectable E protein. **B** Quantification of the LD using ImageJ. The reduction in the LD between zika vs baf + zika is significant at all times. Quantifications are analyzed in more than 200 cells for each condition. **C** Zika transcription as signified by NS1 mRNA is reduced approximately 50% after bafilomycin treatment

We used bafilomycin A1, a very specific inhibitor of autophagy that blocks autophagosome-lysosome fusion by inhibiting vacuolar H^+^ ATPase. We pre-treated MDCK cells with 50 μM bafilomycin A1 for one h before ZIKV infection at MOI of 1. Bafilomycin A1 decreases LD availability by 60–70% after 24 hpi (Fig. 4A, B). Expression of the E protein was markedly reduced in bafilomycin A1 treated cells (Fig. 4A lower panel). Treatment with bafilomycin A1 also reduced viral transcription by nearly 50% (Fig. 4C). Since bafilomycin A1 reportedly disrupts cholesterol esterification in macrophages, [24] we stained infected, bafilomycin-treated cells with filipin III, which localizes to unesterified cholesterol (Additional file 1: Fig. 3S). In Zika-infected cells, unesterified cholesterol disappears by 48 h, and E protein expression is robust. In contrast, in bafilomycin-treated zika-infected cells, unesterified cholesterol accumulates at 48 h and E protein expression is scant. Thus, Bafilomycin A1 inhibits viral expression by blocking cholesterol esterification to form LD.

## Discussion

We propose the cooperation between autophagy and LD that positively contributes to ZIKV replication. Since the ZIKV genome does not have the machinery necessary for lipid synthesis, these lipids are derived from the host cell ER membrane. In our study, infected cells had the most LD seen at 12 hpi and the lowest at 48 hpi (Fig. 1B). Viral transcription was highest at 48 hpi (Fig. 1C). This dependence on lipids for flavivirus life cycle has been documented for several viruses [25–27]. We also report, similarly to Chen et al. [28], an increase in the amount and size of the LD in uninfected neighboring cells compared to infected ones (Additional file 1: Fig. 1S). This phenomenon is shown by several viruses, interpreted by others as indicating either the establishment of intercellular channels within the gap junctions or soluble mediators secreted or taken up by infected cells [29–31]. Thus, ZIKV infection not only directly regulates the lipid metabolism to support the virus life cycle but also induces bystander effects. Variations in LD distribution, viral transcription level changes with time, and the bystander effect all suggest that ZIKV uses host lipids to form its replication complexes and alter the metabolism of surrounding cells to propagate its infection.

LD availability can determine the amount of viral infection. Treatment with ATV reduces the amount of LD (Fig. 2A) and impairs viral transcription, translation, and release (Fig. 2B–D). Statins and their derivatives disrupt infection by ZIKV and other flaviviruses [32, 33]. Cholesterol is significantly redistributed in infected cells with strong colocalization with viral replication (Additional file 1: Fig. 2S). However, with ATV pretreatment, cholesterol is more dispersed. Given that there are no therapeutics or vaccines against ZIKV and that statins are inexpensive with few side effects, they may be a valuable therapeutic against ZIKV.

The interaction between ZIKV and the autophagic pathway is complex and may depend on cell type [19]. In our model, autophagy positively contributes to LD accumulation and ZIKV infection as its inhibition reduces ZIKV E protein expression and synthesis of NS1 RNA (Fig. 3C–E). Previous studies have shown that ZIKV and other flaviviruses increase lipid droplet formation *prior* to the induction of autophagy [34, 35]. Here we show that ZIKV increases autophagy as early as 6 hpi, prior to lipid droplet depletion, which according to our data occurs around 48 hpi (Fig. 3A). Wortmannin pretreatment inhibits early stages of autophagy, and like ATV, reduces LD availability in the cell and overall expression of the virus. Bafilomycin A1 treatment effectively abolished expression of ZIKV E protein (Fig. 4A, lower panel) while significantly decreasing LD (Fig. 4B) and redistributing host cholesterol (Additional file 1: Fig. 2S). We also suggest that bafilomycin blocks cholesterol esterification (Additional file 1: Fig. 3S) and this makes cholesterol unavailable for ZIKV-induced lipid increases, necessary to maintain viral replication. These results highlight the importance of the endosomal-lysosomal compartment for the manipulation of lipids required for the ZIKV lifecycle.

Flavivirus infection results in ER stress, which can promote autophagy and activate transcriptional changes related to the unfolded protein response (UPR). Induction of global ER stress in infected cells using tunicamycin led to almost twice as many lipids compared to Zika alone (Additional file 1: Fig. 4S) However, Salubrinal, an inhibitor of eIF2α dephosphorylation and downregulator of the PERK branch of the UPR, did not change LD compared with ZIKV alone. Thus, global ER stress but not specifically the PERK pathway induces LD in infected cells. Future research should clarify the role of IRE1 and ATF6 pathways to LD in the context of ZIKV infection.

**Fig. 5:**
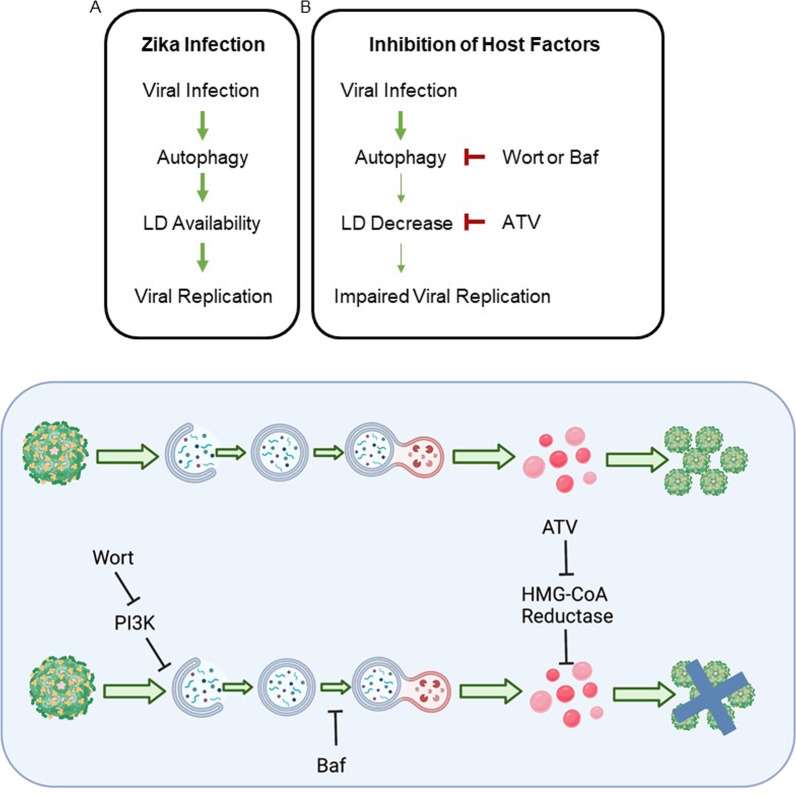
Proposed model. **A** During Zika infection, autophagy increases as early as 6 hpi. Lipid droplet availability increases later in infection around 12 hpi. These events set the stage for uncontrolled viral replication, which peaks at 48 hpi. **B** If early steps in Zika infection are inhibited, such as the increase in autophagy or the increase in lipid droplets, viral replication is compromised. **C** Proposed sequence of events in presence or absence of inhibitors

Taken together, there is a dynamic interplay between ZIKV and host lipids throughout the viral life cycle with autophagy contributing to Zika-induced LD accumulation as summarized in Fig. 5. Inhibition of either ER stress or autophagy alone suppresses, but does not eliminate, the amount of LD. Thus, it can be concluded that the availability of LD depends on several pathways. The induction of LD is a necessary component for replication of the virus, as inhibiting ZIKV-induced increase in lipids with ATV reduces replication (Additional file 1: Fig. S6).

## Conclusion

In conclusion, like other flaviviruses, ZIKV shows patterns of early activation of autophagy. This catabolic process leads to increases in cellular lipids before the virus begins to accumulate. Inhibitors of autophagy that target both the early and late stages of autophagy reduce LD size and viral replication. Our findings on the importance of cholesterol shuffling through the endosomal-lysosomal membranes for ZIKV replication provide one mechanism of many by which ZIKV hijacks cellular lipids.

Statins have not been assessed for their ability to reduce the impact of ZIKV infection in neither randomized clinical trials nor observational studies. We show that ZIKV infection increases LD formation through autophagy and that pharmacological inhibition of LD or autophagy greatly reduces virus replication highlighting lipids as a possible therapeutic target. This is especially important given that there are no current vaccines or therapeutics against ZIKV infection.

## Supplementary Information

**Additional file 1: Figure 1S**: Zika infection modulates lipid droplets in infected and neighboring cells. Lipid droplets are stained redparticles in both mock-infectedand infectedcells after 48 h at MOI 1. **A** Not all cells were infected; those infected display E protein. While the infected cells show variability in the expression of lipid droplets. **B** Overall, there are more lipid droplets in zika infected cells compared to the neighboring uninfected cells. Neighboring cells are defined as those directly next to an E protein expressing cell but which themselves do not have any E protein signal. We refer to them as ZIKV E-. This figure shows ImageJ quantification showing a statistically significant increase when comparing zika infected cellsversus neighboring cells in zika condition. The images shown are representative of the condition. Quantification was achieved by analysis of more than 200 cells for each condition. **C** Pearson Correlation Coefficient for the relationship between virus mRNA, calculated from Ct coefficient, and Oil Red O fluorescence. The correlation is positive for mRNA/ORO, whereas it would be negative for Ct/ORO. **Figure 2S**: Cholesterol is redistributed to sites of ZIKV replication. MDCKs were pretreated with 5 mM ATV for 1 h then infected with ZIKVfor 48 h. Immunofluorescence visualized cholesteroland viral E protein. Beta tubulin was used as a cell marker. Filipin staining is greatest in ZIKV infected cells compared to mock. Following ATV treatment filipin staining is reduced in zika-infected cells. Cholesterol is dispersed throughout the cell in mock conditions but is colocalized with the E protein sites after infection. The images shown are representative of the conditions. **Figure 3S**: Cholesterol is redistributed to sites of ZIKV replication. MDCKs were pretreated with 50 nM bafilomycin for 1 h then infected with ZIKVfor either 12, 24 or 48 h. Immunofluorescence visualized cholesteroland viral E protein. Filipin staining is greatest in ZIKV infected cells compared to mock at 12 hours however with time we see less filipin staining and none at 48 hours. Bafilomycin pretreatment results in filipin staining being always maintained in zika infected cells. Minimal E protein is always detected in bafilomycin pretreated cells. The images shown are representative of the condition. **Figure 4S**: Accumulation of Zika induced lipid droplets is independent of PERK. MDCK cells were pretreated for one hour with either 3 μm salubrinal, an inhibitor of the PERK branch of the UPR, or 3 mM tunicamycinat, a global ER stress inducer. Sal partially suppresses the formation of lipid droplets. However, sal has no effect after zika infection. Tunica dramatically increases lipid droplet number. Compared to zika-infected cells, tunica+zika cells display a near doubling of lipid droplets. These observations were quantified with ImageJ and were statistically significant. Quantifications are performed with more than 200 cells for each condition, in at least 3 independent experiments. **Figure 5S**. Example of Lipid Quantification. Cells marked with asterisks were quantified as Zika Env- for Fig. 1S. Cells circled with a dashed line were quantified as Zika Env+. For mock lipid quantification in Fig. 1S, all lipids were quantified. Nuclear DNA is stained with DAPI. **Figure 6S**. Sources of Antibodies and other Materials.

## Data Availability

All data are held by ZZ and are available on request.

## Abbreviations

ATV: Atorvastatin
Baf: Bafilomycin A1
H: Hour
HPI: Hours post infection
LD: Lipid droplet
MOI: Multiplicity of infection
ORO: Oil Red O
PERK: Protein kinase RNA-like ER kinase
Sal: Salubrinal
Tunica: Tunicamycin
UPR: Unfolded protein response
Wort: Wortmannin
ZIKV: Zika virus

## References

[1] Younger DS (2016). Epidemiology of Zika virus. Neurol Clin.

[2] Dick GWA, Kitchen SF, Haddow AJ (1952). Zika virus (I). Isolations and serological specificity. Trans R Soc Trop Med Hyg.

[3] Gubler DJ, Vasilakis N, Musso D (2017). History and emergence of Zika virus. J Infect Dis.

[4] Calvet G, Aguiar RS, Melo ASO, Sampaio SA, de Filippis I, Fabri A (2016). Detection and sequencing of Zika virus from amniotic fluid of fetuses with microcephaly in Brazil: a case study. Lancet Infect Dis.

[5] Mlakar J, Korva M, Tul N, Popović M, Poljšak-Prijatelj M, Mraz J (2016). Zika virus associated with microcephaly. N Engl J Med.

[6] Brasil P, Calvet GA, Siqueira AM, Wakimoto M, de Sequeira PC, Nobre A (2016). Zika virus outbreak in Rio de Janeiro, Brazil: clinical characterization, epidemiological and virological aspects. PLoS Negl Trop Dis.

[7] do Rosário MS, de Jesus PAP, Vasilakis N, Farias DS, Novaes MAC, Rodrigues SG (2016). Guillain–Barré syndrome after Zika virus infection in Brazil. Am J Trop Med Hyg.

[8] Cortese M, Goellner S, Acosta EG, Neufeldt CJ, Oleksiuk O, Lampe M (2017). Ultrastructural characterization of Zika virus replication factories. Cell Rep.

[9] Jacquemyn J, Cascalho A, Goodchild RE (2017). The ins and outs of endoplasmic reticulum-controlled lipid biosynthesis. EMBO Rep.

[10] Leier HC, Weinstein JB, Kyle JE, Lee JY, Bramer LM, Stratton KG (2020). A global lipid map defines a network essential for Zika virus replication. Nat Commun.

[11] Osuna-Ramos JF, Reyes-Ruiz JM, del Ángel RM (2018). The role of host cholesterol during flavivirus infection. Front Cell Infect Microbiol.

[12] Zhang Z, He G, Filipowicz NA, Randall G, Belov GA, Kopek BG (2019). Host lipids in positive-strand RNA virus genome replication. Front Microbiol.

[13] Shin DW (2020). Lipophagy: molecular mechanisms and implications in metabolic disorders. Mol Cells.

[14] Jackson WT (2015). Viruses and the autophagy pathway. Virology.

[15] Choi Y, Bowman JW, Jung JU (2018). Autophagy during viral infection—a double-edged sword. Nat Rev Microbiol.

[16] Zhou Z, Jiang X, Liu D, Fan Z, Hu X, Yan J (2009). Autophagy is involved in influenza A virus replication. Autophagy.

[17] Lee YR, Lei HY, Liu MT, Wang JR, Chen SH, Jiang-Shieh YF (2008). Autophagic machinery activated by dengue virus enhances virus replication. Virology.

[18] Chan ST, Ou JJ (2017). Hepatitis C virus-induced autophagy and host innate immune response. Viruses.

[19] Chiramel AI, Best SM (2018). Role of autophagy in Zika virus infection and pathogenesis. Virus Res.

[20] Ghosh Roy S. Mechanisms adopted by Dengue-2 viruses to induce autophagy in mammalian cells. Diss Theses Capstone Proj [Internet]. 2018. Available from: https://academicworks.cuny.edu/gc_etds/2919.

[21] Lin L, Ye Y, Zakeri Z (2006). p53, Apaf-1, caspase-3, and -9 are dispensable for Cdk5 activation during cell death. Cell Death Differ.

[22] Episcopio D, Aminov S, Benjamin S, Germain G, Datan E, Landazuri J (2019). Atorvastatin restricts the ability of influenza virus to generate lipid droplets and severely suppresses the replication of the virus. FASEB J Off Publ Fed Am Soc Exp Biol.

[23] Yan Q, Song Y, Zhang L, Chen Z, Yang C, Liu S (2018). Autophagy activation contributes to lipid accumulation in tubular epithelial cells during kidney fibrosis. Cell Death Discov.

[24] Furuchi T, Aikawa K, Arai H, Inoue K (1993). Bafilomycin A1, a specific inhibitor of vacuolar-type H(+)-ATPase, blocks lysosomal cholesterol trafficking in macrophages. J Biol Chem.

[25] Gillespie LK, Hoenen A, Morgan G, Mackenzie JM (2010). The endoplasmic reticulum provides the membrane platform for biogenesis of the flavivirus replication complex. J Virol.

[26] Martín-Acebes MA, Vázquez-Calvo Á, Saiz JC (2016). Lipids and flaviviruses, present and future perspectives for the control of dengue, Zika, and West Nile viruses. Prog Lipid Res.

[27] Perera R, Riley C, Isaac G, Hopf-Jannasch AS, Moore RJ, Weitz KW (2012). Dengue virus infection perturbs lipid homeostasis in infected mosquito cells. PLOS Pathog.

[28] Chen Q, Gouilly J, Ferrat YJ, Espino A, Glaziou Q, Cartron G (2020). Metabolic reprogramming by Zika virus provokes inflammation in human placenta. Nat Commun.

[29] Palmer DR, Sun P, Celluzzi C, Bisbing J, Pang S, Sun W (2005). Differential effects of dengue virus on infected and bystander dendritic cells. J Virol.

[30] Zhou H, Ivanov VN, Gillespie J, Geard CR, Amundson SA, Brenner DJ (2005). Mechanism of radiation-induced bystander effect: role of the cyclooxygenase-2 signaling pathway. Proc Natl Acad Sci.

[31] Kofahi HM, Taylor NGA, Hirasawa K, Grant MD, Russell RS (2016). Hepatitis C virus infection of cultured human hepatoma cells causes apoptosis and pyroptosis in both infected and bystander cells. Sci Rep.

[32] Martín-Acebes MA, Jiménez de Oya N, Saiz JC (2019). Lipid metabolism as a source of druggable targets for antiviral discovery against Zika and other flaviviruses. Pharmaceuticals.

[33] Españo E, Nam JH, Song EJ, Song D, Lee CK, Kim JK (2019). Lipophilic statins inhibit Zika virus production in Vero cells. Sci Rep.

[34] Heaton NS, Randall G (2010). Dengue virus-induced autophagy regulates lipid metabolism. Cell Host Microbe.

[35] Zhang J, Lan Y, Li MY, Lamers MM, Fusade-Boyer M, Klemm E (2018). Flaviviruses exploit the lipid droplet protein AUP1 to trigger lipophagy and drive virus production. Cell Host Microbe.

