# Supplementary material for: Zika virus triggers autophagy to exploit host lipid metabolism and drive viral replication
Source: Cell Commun Signal. 2023 May 19;21:114. doi: 10.1186/s12964-022-01026-8 (PMC10199480; doi:10.1186/s12964-022-01026-8)
Supplement: Supplementary file 2 — Additional file 1: Figure 1S: Zika infection modulates lipid droplets in infected and neighboring cells. Lipid droplets are stained redparticles in both mock-infectedand infectedcells after 48 h at MOI 1. A Not all cells were infected; those infected display E protein. While the infected cells show variability in the expression of lipid droplets. B Overall, there are more lipid droplets in zika infected cells compared to the neighboring uninfected cells. Neighboring cells are defined as those directly next to an E protein expressing cell but which themselves do not have any E protein signal. We refer to them as ZIKV E-. This figure shows ImageJ quantification showing a statistically significant increase when comparing zika infected cellsversus neighboring cells in zika condition. The images shown are representative of the condition. Quantification was achieved by analysis of more than 200 cells for each condition. C Pearson Correlation Coefficient for the relationship between virus mRNA, calculated from Ct coefficient, and Oil Red O fluorescence. The correlation is positive for mRNA/ORO, whereas it would be negative for Ct/ORO. Figure 2S: Cholesterol is redistributed to sites of ZIKV replication. MDCKs were pretreated with 5 mM ATV for 1 h then infected with ZIKVfor 48 h. Immunofluorescence visualized cholesteroland viral E protein. Beta tubulin was used as a cell marker. Filipin staining is greatest in ZIKV infected cells compared to mock. Following ATV treatment filipin staining is reduced in zika-infected cells. Cholesterol is dispersed throughout the cell in mock conditions but is colocalized with the E protein sites after infection. The images shown are representative of the conditions. Figure 3S: Cholesterol is redistributed to sites of ZIKV replication. MDCKs were pretreated with 50 nM bafilomycin for 1 h then infected with ZIKVfor either 12, 24 or 48 h. Immunofluorescence visualized cholesteroland viral E protein. Filipin staining is greatest in ZIKV infecte [file 12964_2022_1026_MOESM1_ESM.pptx]

## Slide 1
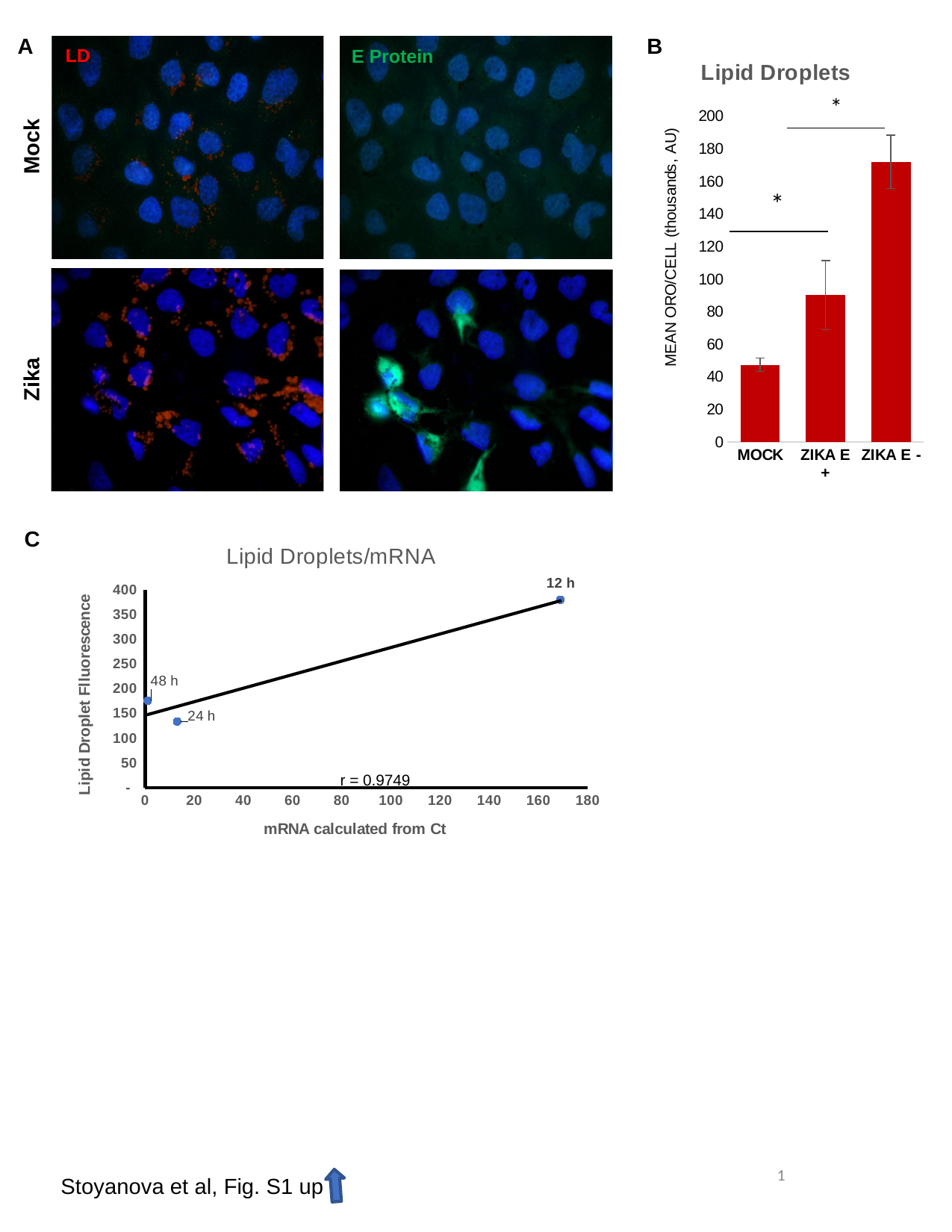

A
B
LD
E Protein
### Chart: Lipid Droplets
| Category | average |
|---|---|
| MOCK | 47.4402079878049 |
| ZIKA E + | 90.23780678571433 |
| ZIKA E - | 172.09683554687498 |*
*
Mock
Zika
C
### Chart: Lipid Droplets/mRNA
| Category | |
|---|---| r = 0.9749
1
Stoyanova et al, Fig. S1 up

## Slide 2
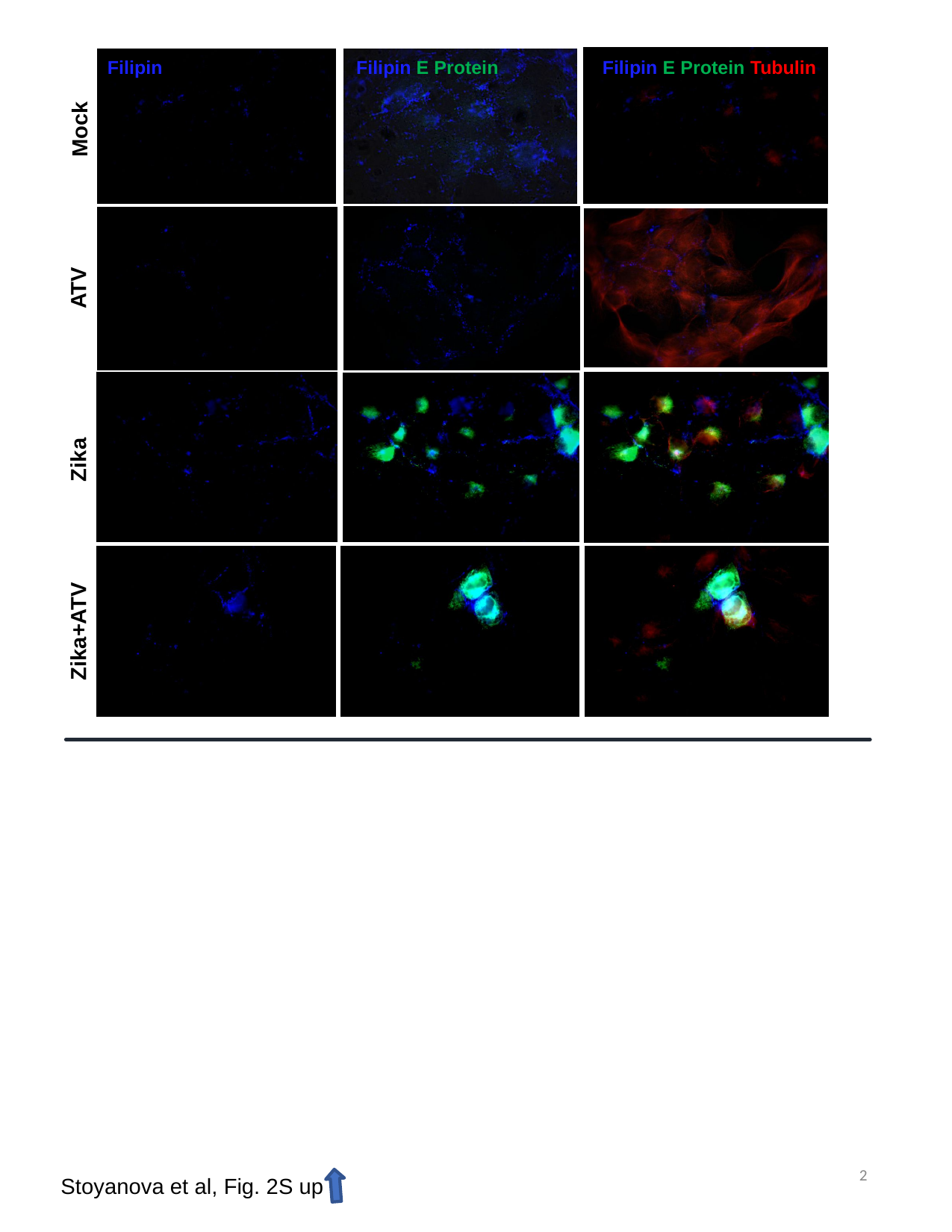

Filipin E Protein Tubulin
Filipin
Filipin E Protein
Mock
ATV
Zika
Zika+ATV
2
Stoyanova et al, Fig. 2S up

## Slide 3
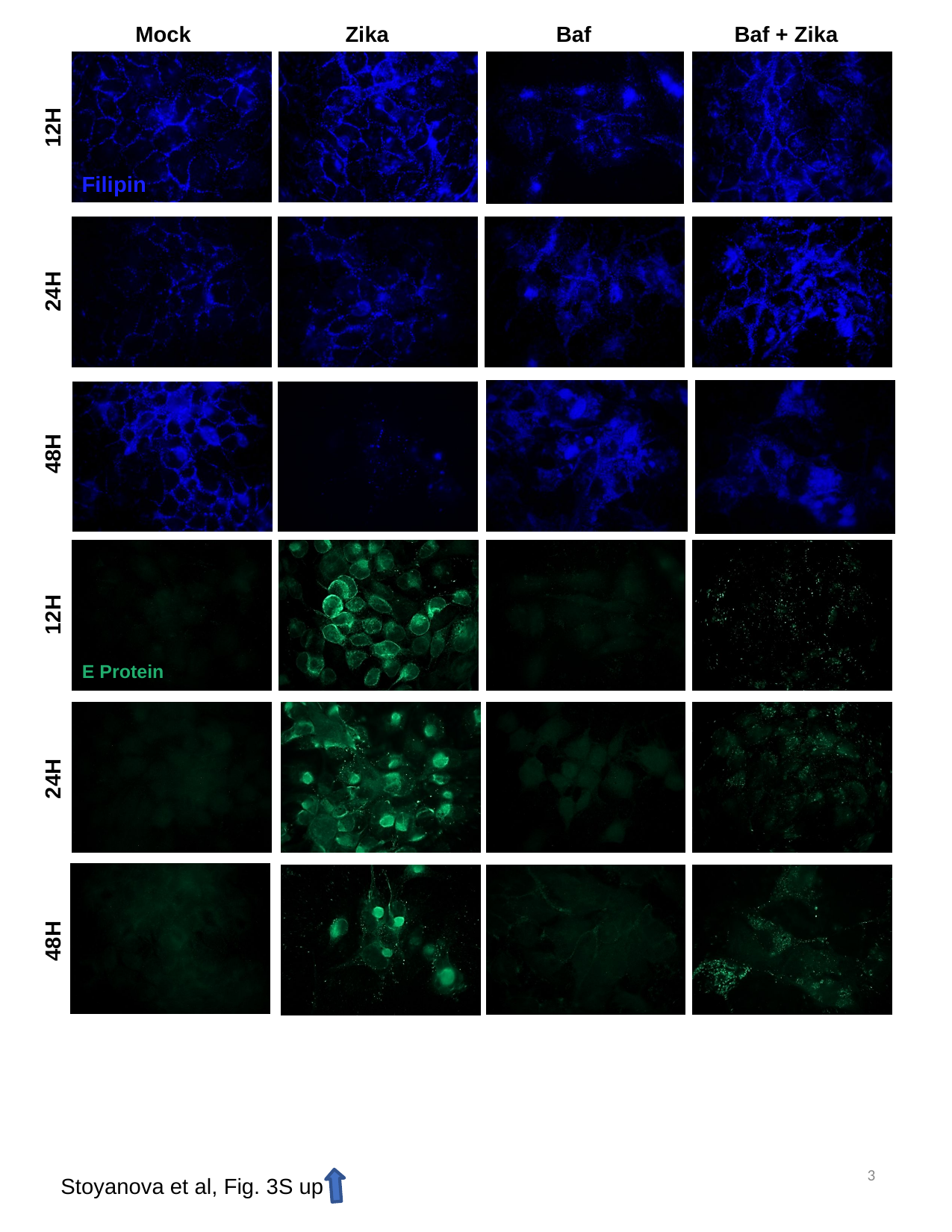

Mock
Zika
Baf
Baf + Zika
12H
Filipin
24H
48H
12H
E Protein
24H
48H
3
Stoyanova et al, Fig. 3S up
3

## Slide 4
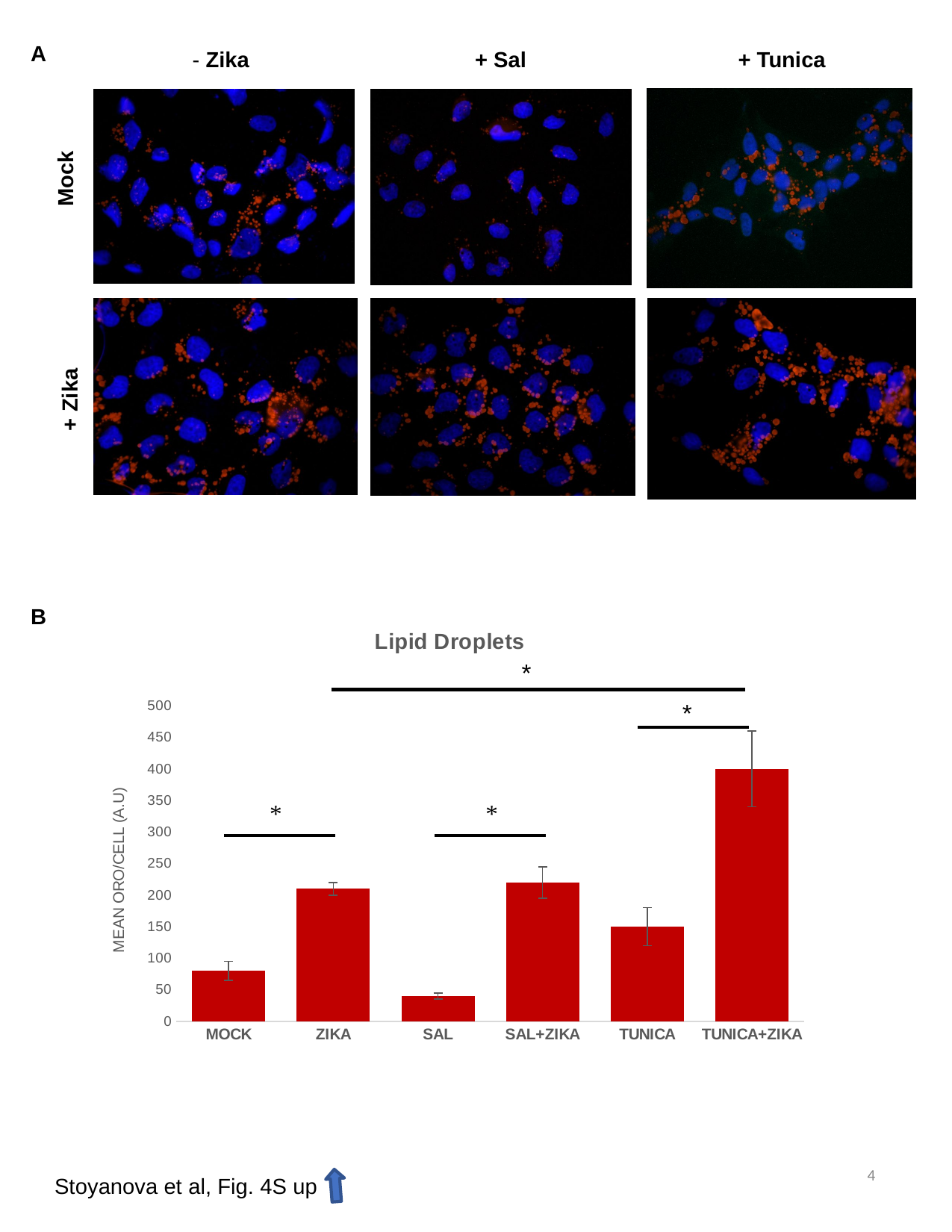

A
- Zika
+ Sal
+ Tunica
Mock
+ Zika
B
### Chart: Lipid Droplets
| Category | |
|---|---|
| MOCK | 80.0 |
| ZIKA | 210.0 |
| SAL | 40.0 |
| SAL+ZIKA | 220.0 |
| TUNICA | 150.0 |
| TUNICA+ZIKA | 400.0 |4
Stoyanova et al, Fig. 4S up

## Slide 5
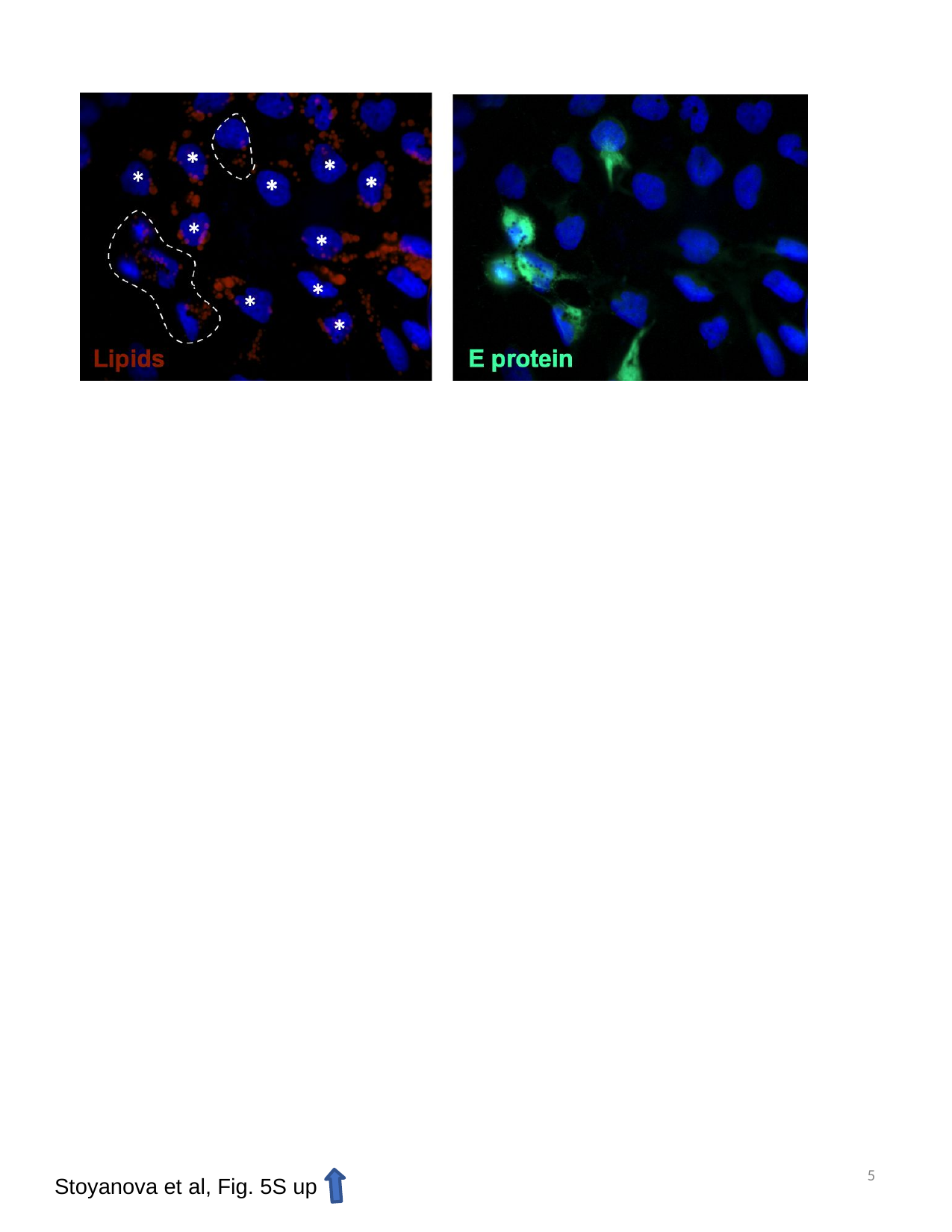

5
Stoyanova et al, Fig. 5S up

## Slide 6
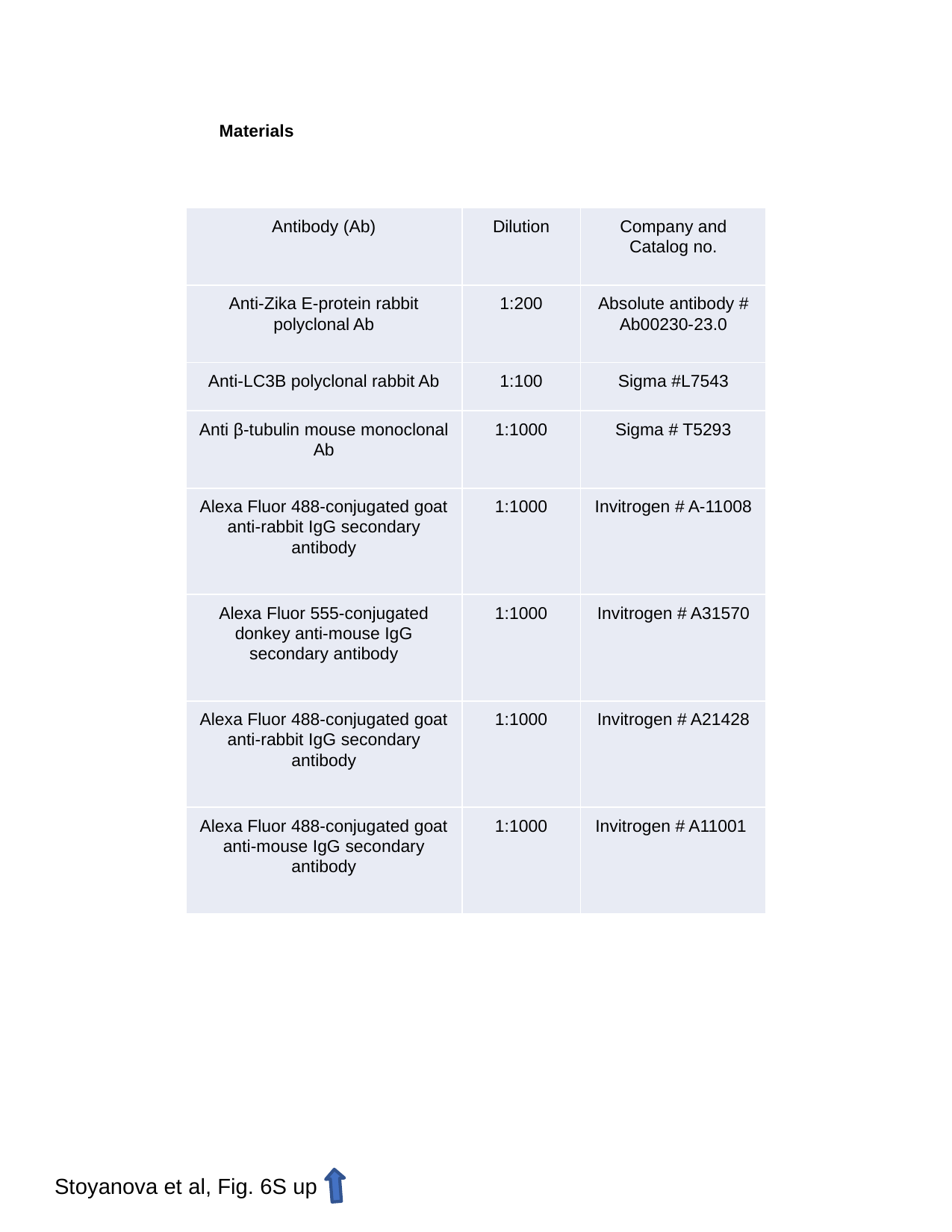

Materials
| Antibody (Ab) | Dilution | Company and Catalog no. |
| --- | --- | --- |
| Anti-Zika E-protein rabbit polyclonal Ab | 1:200 | Absolute antibody # Ab00230-23.0 |
| Anti-LC3B polyclonal rabbit Ab | 1:100 | Sigma #L7543 |
| Anti β-tubulin mouse monoclonal Ab | 1:1000 | Sigma # T5293 |
| Alexa Fluor 488-conjugated goat anti-rabbit IgG secondary antibody | 1:1000 | Invitrogen # A-11008 |
| Alexa Fluor 555-conjugated donkey anti-mouse IgG secondary antibody | 1:1000 | Invitrogen # A31570 |
| Alexa Fluor 488-conjugated goat anti-rabbit IgG secondary antibody | 1:1000 | Invitrogen # A21428 |
| Alexa Fluor 488-conjugated goat anti-mouse IgG secondary antibody | 1:1000 | Invitrogen # A11001 |
Stoyanova et al, Fig. 6S up
